# A two-hybrid system reveals previously uncharacterized protein–protein interactions within the *Helicobacter pylori* NIF iron–sulfur maturation system

**DOI:** 10.1038/s41598-021-90003-1

**Published:** 2021-05-24

**Authors:** Stéphane L. Benoit, Stephanie Agudelo, Robert J. Maier

**Affiliations:** 1 Department of Microbiology, The University of Georgia, 30602 Athens, Georgia; 2 Center for Metalloenzyme Studies, The University of Georgia, 30602 Athens, Georgia

**Keywords:** Biochemistry, Microbiology

## Abstract

Iron–sulfur (Fe–S) proteins play essential roles in all living organisms. The gastric pathogen *Helicobacter pylori* relies exclusively on the NIF system for biosynthesis and delivery of Fe–S clusters. Previously characterized components include two essential proteins, NifS (cysteine desulfurase) and NifU (scaffold protein), and a dispensable Fe–S carrier, Nfu. Among 38 proteins previously predicted to coordinate Fe–S clusters, two proteins, HP0207 (a member of the Nbp35/ApbC ATPase family) and HP0277 (previously annotated as FdxA, a member of the YfhL ferredoxin-like family) were further studied, using a bacterial two-hybrid system approach to identify protein–protein interactions. ApbC was found to interact with 30 proteins, including itself, NifS, NifU, Nfu and FdxA, and alteration of the conserved ATPase motif in ApbC resulted in a significant (50%) decrease in the number of protein interactions, suggesting the ATpase activity is needed for some ApbC-target protein interactions. FdxA was shown to interact with 21 proteins, including itself, NifS, ApbC and Nfu, however no interactions between NifU and FdxA were detected. By use of cross-linking studies, a 51-kDa ApbC-Nfu heterodimer complex was identified. Attempts to generate *apbC* chromosomal deletion mutants in *H. pylori* were unsuccessful, therefore indirectly suggesting the *hp0207* gene is essential. In contrast, mutants in the *fdxA* gene were obtained, albeit only in one parental strain (26695). Taken together, these results suggest both ApbC and FdxA are important players in the *H. pylori* NIF maturation system.

## Introduction

Iron–sulfur (Fe–S) clusters are ubiquitous and essential in all domains of life, since they are cofactors for proteins playing vital roles in the cell, such as in DNA repair, cofactor biosynthesis, regulation of gene expression, respiration, photosynthesis, and metabolism (reviewed in^1,2^). For instance, the list includes Fe–S proteins belonging to eukaryotic mitochondrial respiratory chains or bacterial electron transport chain (complex I), eukaryotic and prokaryotic aconitases, ferredoxins, *S*-adenosylmethionine (SAM) radical enzymes, nitrogenase, and regulatory proteins such as SoxR, FNR and IRP^1,2^. In humans, diseases caused by defects in Fe–S cluster biogenesis include Friedreich’s ataxia, GLRX5-deficient sideroblastic anemia, and several mitochondrial diseases (most of which are rare, but fatal)^3^. Synthesis of Fe–S clusters is a complex, multistep process that requires the combined work of several proteins. Three distinct systems for Fe–S cluster assembly are known: ISC (*i*ron-*s*ulfur *c*luster, recently reviewed in^4^), NIF (*ni*trogen *f*ixation) and SUF (*s*ulfur *u*tilization *f*actors). Bacteria can have one, two or all three assembly systems. Although the three Fe–S cluster biosynthetic pathways have distinct machineries, two core components are conserved among them. First, it is widely accepted that L-Cysteine provides the inorganic sulfur, via a pyridoxal-5’-phosphate-dependent cysteine desulfurase, such as Csd A/E^5^, IscS^6^, NifS^7^ or Suf S/E^8,9^, depending on the Fe–S maturation system. Second, all Fe–S clusters are assembled on a scaffold protein, such as SufB^10^, IscU^11^, or NifU^12^, more specifically its amino-acid terminal domain^13^; or alternatively, on a “A-type” scaffold, *e.g.* IscA^14^, ^Nif^IscA^15^ or SufA^9^. It is still debated whether the ferrous (Fe^2+^) ions come from free Fe^2+^ pools in the cell or from dedicated proteins. Regarding the latter, previous studies have found a role in iron supply for proteins such as CyaY and YtfE^16,17^. Indeed, the *Escherichia coli* CyaY, an iron-binding protein homologous to eukaryotic frataxin, was found to interact with IscS, leading eventually to [2Fe-2S] clusters assembly on the IscU scaffold protein^16^. Likewise, there is evidence that YtfE, also known as “RIC” (Repair of Iron Centers), can reconstitute Fe–S centers in vitro, either in spinach ferredoxin or within *E. coli* IscU^17^. Once Fe–S clusters have been assembled on scaffold proteins, intact Fe–S clusters are transferred to recipient proteins, either directly or via intermediate carrier proteins. Those include *A*-*t*ype *c*arrier (ATC) proteins, such as the ones cited above (IscA, ^Nif^IscA, SufA,)^15,18^, as well as ErpA^19^; other carriers include monothiol glutaredoxins^20–22^, and Nfu proteins, sometimes called NifU-like proteins, because they contain a conserved domain (CXXC) that can also be found in the C-terminal domain of NifU^23^. The conserved cysteines of the CXXC domain have been shown to bind [2Fe–2S] and/or [4Fe–4S]^24,25^.

Among the three systems described above, the NIF system is used by nitrogen-fixing bacteria for the maturation of Fe–S nitrogenase (reviewed in^1^). Unexpectedly, it is also the sole Fe–S maturation system used by members of the ε-proteobacteria family, such as *Helicobacter* or *Campylobacter* species*,* even though none of those species (*Helicobacter pylori* included) contains nitrogenase. Both NifS and NifU proteins can be found in *H. pylori*: those are HP0220 and HP0221, respectively, as annotated in *H. pylori* strain 26695^26^(Table 1). Based on a previously published study, the *H. pylori* NIF maturation system (as minimally defined by the bicistronic *nifS*-*nifU* operon) is complete and versatile, since it can substitute for both SUF and ISC systems in *E. coli*^27^. Previous attempts to disrupt *nifS* or *nifU* chromosomal copies in *H. pylori* were unsuccessful, suggesting that *nifS* and *nifU* are both essential genes^28^. However, conditional *nifU* mutants have recently been constructed in our laboratory^29^. As expected, those mutants were more sensitive to oxidative stress compared to wild-type (WT) strains^29^. Besides NifS, *H. pylori* possess a NifS-like homolog (HP0405), whose role is yet unknown. The *hp0405* gene locus has been successfully disrupted (suggesting it is not essential) without any apparent phenotype; a hot spot for recombination, this site has been used for complementation purposes in more than fifteen studies, conducted in our lab and others (for instance see^28,30–32^).

**Table 1:** Main components of the *H. pylori* NIF system.

HP number^a^	ID	Uniprot entry	Name (proposed function)	Essential?^b^	Amino acids (size in kDa)	Interactions Fe–S prots (number of interactions)^c^
HP0220	NifS	25008	l-cysteine desulfurase (sulfur donor)	Yes	387 (42.4)	Yes (6)
HP0405	NifS-like	25161	NifS-like (unknown)	No	440 (48.5)	Unknown
HP0221	NifU	25009	Main scaffold protein (Fe–S synthesis)	Yes	326 (36.4)	Yes (29)
HP1492	Nfu	26025	Nfu-type (Fe–S carrier)	No	89 (10.1)	Yes (15)
HP0207	ApbC/Mrp	24999	Member of Nbp35 ATPase (unknown; Fe–S carrier?)	Yes	368 (40)	Unknown^e^
HP0277	FdxA	25054	Ferredoxin (unknown; Fe–S carrier?)	Yes/no^d^	84 (9.5)	Unknown^e^

^a^HP number refers to strain 26695^26^.

^b^Based on chromosomal mutagenesis attempts (as found in this study, or as reported by^28,29,34^).

^c^As reported by^29^.

^d^*H. pylori* strain specific (as found in this study, and as reported by^34^).

^e^Before the current study.

Whereas *H. pylori* does not contain the ^Nif^IscA ATC commonly found in the *nif* operons of nitrogen-fixing bacteria^26^, the gastric pathogen has a Nfu-type protein (HP1492) that shares similarity with the C-terminus of NifU^27,29^. Purified recombinant *Hp*Nfu is capable of binding either [2Fe–2S] or [4Fe–4S] clusters^29^. Furthermore, *nfu* deletion mutants were successfully generated in several wild-type strains, indicating the *hp1492*/*nfu* gene is not essential^29^. The *nfu* mutants displayed (i) increased oxidative stress sensitivity, (ii) higher hydrogenase activity, (iii) lower aconitase activity and (iv) reduced colonization of the mouse mucosa^29^. Hence, the Nfu/HP1492 protein plays important roles in the cell, even though it is dispensable, in contrast to NifU and NifS.

Based on sequence analysis, *H. pylori* possess (at least) 36 proteins predicted to contain Fe–S clusters, besides NifU and Nfu^29^. Using a Bacterial Adenylate Cyclase Two-Hybrid (BACTH) system^33^, in vivo protein–protein interactions were found between (i) NifS and NifU; (ii) NifS and Nfu; (iii); NifU and Nfu; (iv) each of the three aforementioned proteins and numerous proteins among the 36 putative Fe–S proteins^29^. Furthermore, a (NifS-NifU)_2_ heterodimeric complex was identified by size exclusion chromatography (SEC)^29^. Among the 36 putative Fe–S cluster-containing proteins, two proteins, HP0207 (a member of the Nbp35/ApbC ATPase family) and HP0277 (previously annotated as FdxA, a member of the ferredoxin-like YfhL family) stand out; firstly, they were among very few proteins shown to interact with the cysteine desulfurase NifS^29^, suggesting they might play a role as stand-alone scaffold protein, thus substituting for NifU; secondly, based on their amino-acid sequence and homology to proteins previously identified in other organisms as Fe–S carriers, each could play a similar role (*e.g.* Fe–S carrier) in *H. pylori*. In the present study, we attempted to construct mutants in either gene (*apbC* or *fdxA*). The *apbC* (*hp0207*) gene appears essential in *H. pylori*, since repeated attempts to recover mutants in three different parental strains failed. In contrast, we were able to disrupt the *fdxA* (*hp0277*) gene in strain 26695 (but not in other strains), in agreement with a previous study demonstrating the essentiality of *fdxA* is background-dependent^34^. The BACTH system was used to decipher the network of interactions between ApbC or FdxA and *H. pylori* proteins predicted to contain Fe–S clusters. In addition, physical interaction between purified recombinant ApbC and Nfu proteins was confirmed, using a crosslinking approach. Finally, the effect of mutations in the ApbC ATPase conserved motif on protein–protein interactions was analyzed. Taken together, our results reveal that ApbC and (to a lesser extent) FdxA play a role in the *H. pylori* NIF maturation system, probably as Fe–S cluster carriers, although this remains to be experimentally shown.

## Results and discussion

### HP0207, a protein belonging to the Nbp35/ApbC family, is essential in *H. pylori*

**Figure 1:**
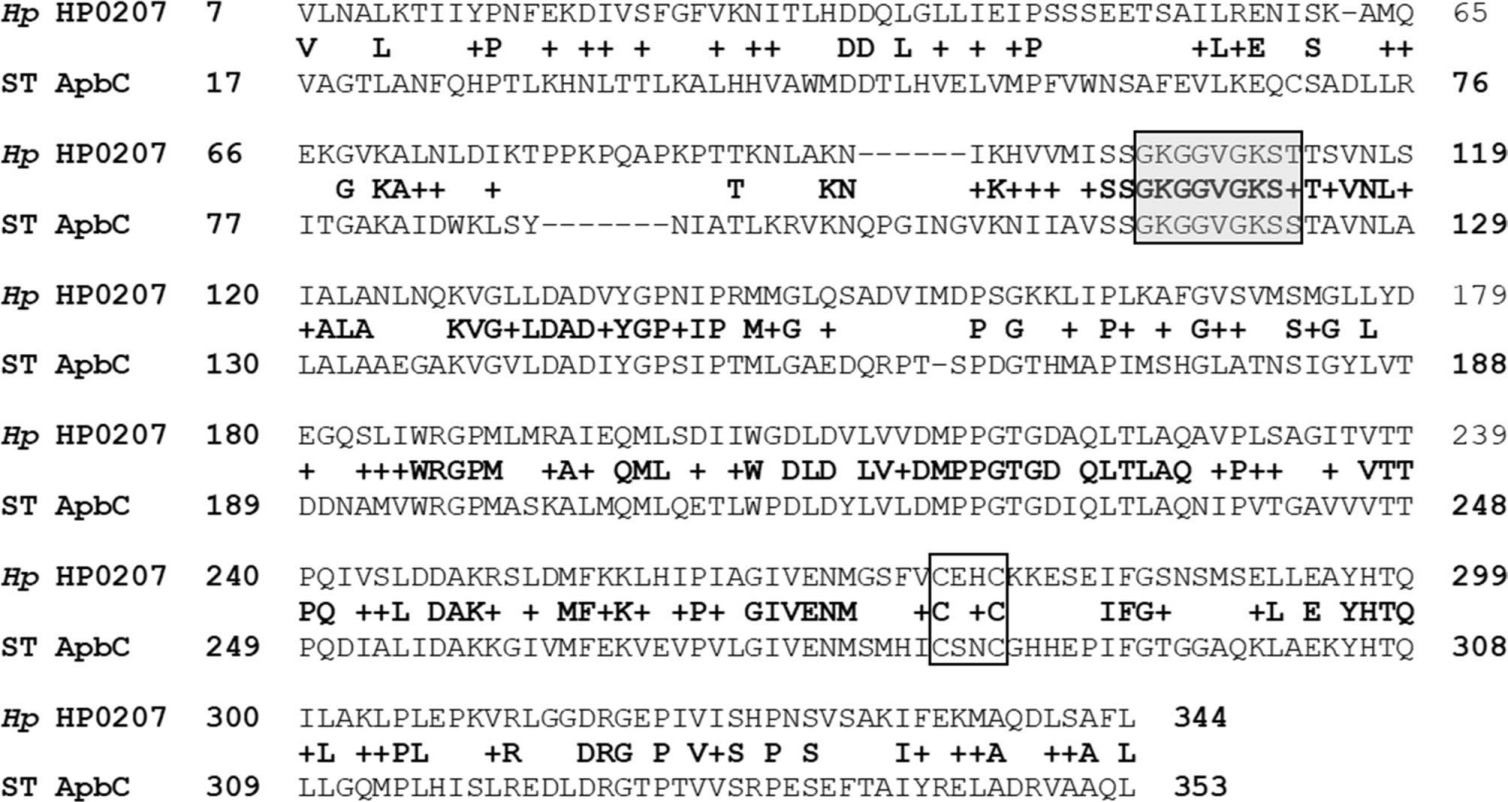
Sequence alignment of *H. pylori* HP0207 and *S.* Typhimurium ApbC. *H. pylori* HP0207 shares 38% identity and 59% similarity with *S.* Typhimurium ApbC. A deviant Walker A motif involved in ATPase activity is shown in the shaded box. A conserved CXXC motif required for Fe–S binding and in vivo transfer is shown in the white box. The sequence alignment was done using BlastP (https://blast.ncbi.nlm.nih.gov/).

Analysis of the amino-acid sequence of *H. pylori* HP0207 suggests the protein belongs to the Nbp35/ApbC family, a well-studied class of proteins with ATPase activity, known to bind and transfer Fe–S clusters in vitro as well as in vivo, as shown in bacteria (*Salmonella enterica* serovar Typhimurium ApbC)^35,36^, archaea (*Methanococcus maripaludis* MMP0704)^37,38^ and eukarya (*Sacharomyces cerevisiae* Cfd1 and Nbd35)^39,40^. A sequence alignment of HP0207 and *S.* Typhimurium ApbC is shown in Fig. 1. Importantly, a Walker A motif (GKGGhGKS/T, with h being hydrophobic residue), described as “deviant” because it contains two conserved lysine residues instead of one, is present in both HP0207 and *S*.T. ApbC (Fig. 1, shaded box). This motif has previously been shown to be involved in ATP binding and hydrolysis in *Salmonella*^36^. In addition, both HP0207 and *S*.T. ApbC proteins contain a ferredoxin-type CxxC motif (Fig. 1, white box). Such conserved motif has been shown to bind [4Fe-4S] clusters (this usually happens at the interface of homo or heterodimeric proteins) in various organisms/proteins, such as *S*.Typhimurium/ ApbC^35^, *Desulfovibrio/* MrpORP^41^, or *S. cerevisiae*/Cfd1 and Nbp35^42,43^. *H. pylori* NifU and Nfu also have a conserved ferredoxin CxxC motif, thought to be involved in coordinating [4Fe–4S]^28,29^. The actual transfer of [4Fe–4S] clusters from members of the Nbp35/ApbC family onto target proteins has been demonstrated in vitro using various models. For instance, Boyd et al. provided evidence of [4Fe–4S] cluster transfer from *S*. Typhimurium holo-ApbC to *S. cerevisiae* apo-isopropylmalate isolomerase (Leu1)^35^; likewise, Zhao et al. recently showed transfer of [4Fe-4S] clusters between *M. maripaludis* holo-MMP0704 and *E. coli* apo-aconitase proteins^38^. A survey of 100 sequenced *H. pylori* genomes showed that the ApbC sequence is highly conserved in *H. pylori* (98.37–100% identity). As expected, both the deviant Walker A motif and the ferredoxin-type CxxC motif are highly conserved in all *H. pylori* genomes (data not shown). At this stage, it is worth noting that the *hp0207* (*apbC*) gene is wrongly annotated in many *H. pylori* genome sequences and several databases (*e.g.* Genbank, Biocyc), with a putative misassigned ATG start codon located 132 bp upstream of the actual start (see Uniprot for the actual start).

 In the present study, we aimed at generating *apbC* deletion mutants in *H. pylori* to determine whether the *H. pylori* ApbC protein plays a role in coordinating and transferring Fe–S clusters, like its *Salmonella* ApbC homolog. We used a (deletion-insertion) mutagenesis method successfully used in our lab to generate numerous mutants, including most recently *nfu* mutants^29^. However, repeated attempts to generate *hp0207* mutants in three different *H. pylori* parental strains (*i.e.* 26695, 43504 and X47) were unsuccessful, suggesting the *apbC* gene is essential in *H. pylori*, as previously observed for *nifS* and *nifU*^28,29^. Additional experimental approaches will be needed to confirm the essentiality of the *H. pylori apbC* gene. For instance, generating conditional *apbC* mutants or constructing mero-diploids *apbC* strains, as previously done for other essential *H. pylori* genes, such as *nifU* and *tatC *^28,29,44^, could be informative. Nevertheless, a likely reason for the failure to obtain *apbC* mutants (or *nifS* and *nifU* mutants) is the fact that *H. pylori* only relies on the NIF system to deliver Fe–S clusters to proteins, some of which perform vital roles in the cell; for instance components of the essential NADH:ubiquinone respiratory pathway, such as NuoB (HP1261), Nqo3 (HP1266) and Nqo9 (HP1268). In contrast, mutants in Apbc/Nbp35 genes have been successfully generated in microorganisms that possess multiple overlapping Fe–S cluster maturation pathways (*e.g.* ISC and SUF). For instance, *S.* Typhimurium *apbC* and M. *maripaludis* *mmp0704* mutants have been reported^38,45^ and genetic studies indicated that ApbC has a degree of functional redundancy with the IscU scaffold protein^46^.

### HP0277/FdxA, a ferredoxin-like protein that belongs to the YfhL family, is essential in some but not all *H. pylori* strains

**Figure 2:**
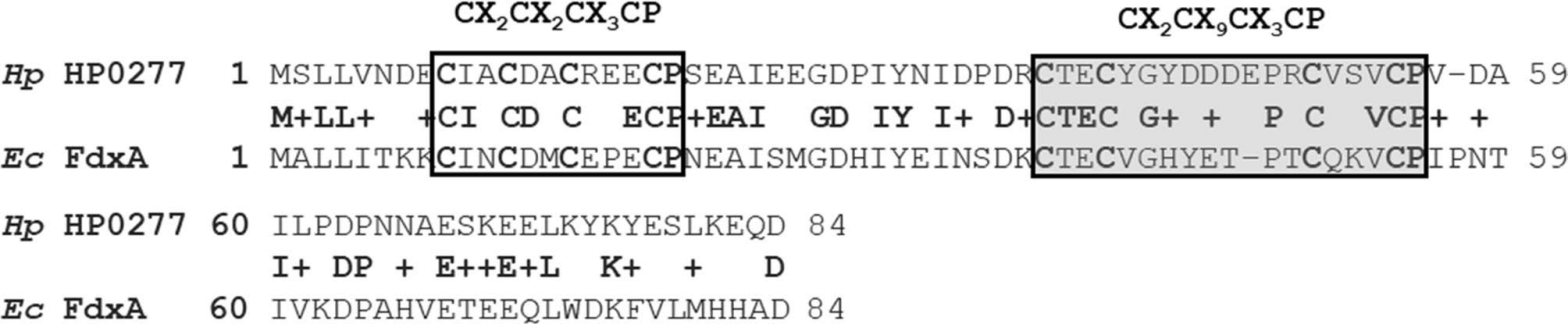
Sequence alignment of *H. pylori* HP0277 and *E. coli* Fdx. *H. pylori* HP0207 shares 45% identity and 64% similarity with *E. coli* Fdx. A conserved ferredoxin CX_2_CX_2_CX_3_C(P) motif is shown in the white box and a ferredoxin-like CX_2_CX_9_CX_3_CP motif (hallmark of the YfhL family) is shown in the shaded box. The sequence alignment was done using BlastP (https://blast.ncbi.nlm.nih.gov/).

Among the 38 proteins previously predicted to contain Fe–S clusters (including NifU and Nfu), eight proteins have [4Fe–4S]-binding ferredoxin-like domains, based on the presence of conserved CX_2_CX_2_CX_3_C(P) motifs^29^. However, the small (84 amino acid long) HP0277 protein, previously annotated as FdxA^34^, stands out: in addition to the aforementioned canonical domain, FdxA also possesses a CX_2_CX_9_CX_3_CP domain (Fig. 2). As such, it belongs to the YfhL family of [4Fe-4S] dicluster ferredoxins. Yfhl ferredoxins are found in a wide range of bacteria, including all -proteobacteria, many environmental nitrogen-fixing bacteria that rely on the NIF maturation system for [4Fe-4S] homeostasis, as well as γ-proteobacteria, such as *E. coli* and S. Typhimurium.

Mutants in the *yfhL*/*fdxA* gene have been generated and characterized in various organisms, including in *E. coli*, *C. jejuni* and *H. pylori*^34,47,48^. In *C. jejuni*, the FdxA homolog (39% identity with HP0277) is iron-induced and plays a role in oxidative stress resistance^48^. In *E. coli*, FdxA has been shown to be required for incorporation of [4Fe–4S] clusters into two hydrogenases, Hyd-1 and Hyd-2. Consequently, *E. coli fdxA* mutants are deficient in hydrogen (H_2_)-oxidizing hydrogenase activity^47^. In the present study, attempts were made to disrupt the *hp0277* gene, using a previously described method^29^. Whereas we could not obtain any mutant in strains 43504 or X47, *fdxA* mutants were isolated in strain 26695. The concomitant deletion of the *fdxA* gene and the insertion of the *cat* marker in the chromosome of strain 26695 were confirmed by PCR followed by agarose gel analysis (data not shown). The fact that we could only generate *fdxA* mutants in some, but not all, *H. pylori* strains was not unexpected, as similar results had previously been reported. Indeed, Mukhopadhyay et al. found a correlation between the metronidazole (Mtz) resistance/sensitivity status of *H. pylori* strains, and their readiness to have *fdxA* inactivated^34^. Thus, the *fdxA* gene can usually be inactivated in strains expressing the *frxA* nitroreductase gene (*hp0642* in strain 26695), *i.e.*, Mtz^S^ strains, such as strain 26695; whereas *fdxA* can rarely (or never) be inactivated in strains with low or no *frxA* expression, *i.e.*, Mtz^R^ strains, such as strains 43504 and X47^34^. In fact, the authors of the study showed that *fdxA* could be disrupted in some Mtz^R^ strains, but only after experimental inactivation of *frxA*; these strains then became Mtz^S^. In summary, contrary to other members of the *H. pylori* NIF maturation system, such as *nifS*, *nifU* and *apbC*, the *hp0277* gene encoding for the ferredoxin-like FdxA is dispensable, at least under certain (genetic background) conditions.

The 26695 *fdxA* mutant was further studied; more specifically, we hypothesized hydrogenase activity would be affected in this mutant. Indeed, *H. pylori* possesses one (H_2_)-oxidizing heterotrimeric hydrogenase, encoded by the *hp0631–hp0632–hp0633* genes in strain 26695 (previously known as HydABC, and recently renamed HynABC^49^). The enzyme is predicted to contain a [4Fe–4S] cluster^29^. We hypothesized the hydrogenase activity would be affected in the *fdxA* mutant, based on (weak) protein–protein interaction between FdxA and the [4Fe–4S] subunit HP0631 (as shown by BACTH, see below), and deficiency in H_2_-oxidizing hydrogenase activity, as previously reported for the *E. coli fdxA* mutant^47^. Whole cell H_2_-uptake hydrogenase assays were carried out with *H. pylori* WT and *fdxA* mutant cells, using a previously described method^50^. Surprisingly*,* hydrogenase activity in the *fdxA* mutant was comparable to that of the WT (71 ± 15 and 59 ± 9 nmoles H_2_ per min per 10^9^ cells, respectively). Therefore, it does not appear that the FdxA protein is required for hydrogenase [4Fe-4S] cluster. Further characterization of the *fdxA* mutant will be needed to fully understand the physiological role of the ferredoxin protein in the gastric pathogen.

### Use of BATCH system reveals novel interactions involving ApbC and FdxA

To investigate protein–protein interactions between ApbC or FdxA, and all proteins identified as putative Fe–S clusters-containing proteins, we used a bacterial (*E. coli*) adenylate cyclase-based two-hybrid system (BACTH)^33^. This system was previously used in our lab to decipher interactions between *H. pylori* NifS, NifU, Nfu, and 36 proteins predicted to coordinate Fe–S clusters, including ApbC and FdxA^29^. Although *E. coli* contains an ApbC homolog, it has limited homology with the *H. pylori* ApbC protein (38% identity / 60% similarity). Thus, we did not anticipate its presence in the background strain to be a problem for using *H. pylori* ApbC in the BACTH system. Briefly, interaction between plasmid-encoded T18 and T25 peptides is required to turn on cAMP-CRP dependent operons, such as *lac* or *mal* operons. The activation can be monitored on screening media, such as MacConkey-Mal (MC-Mal) or LB-X-Gal, or on a selection medium, such as M63-Mal. In the present study, *apbC* (*hp0207*) was cloned in plasmid pKT25, thus generating a T25-ApbC chimeric protein; *fdxA* (*hp0277*) was cloned in plasmid pKNT25, thus producing a FdxA-T25 fusion protein. Both genes (*e.g. apbC* and *fdxA*), as well as *nifS*, *nifU*, *nfu* and 34 other genes encoding for putative Fe–S-containing proteins, were previously cloned in plasmid pUT18C, generating T18-target fusion proteins^29^. After *E. coli cya* mutants (BTH101, Table S1) were co-transformed with both pK(N)T25 and pUT18C derivatives, cells were spotted on MC-Mal and LB-X-Gal plates, which were then incubated for 36 to 48 h at 30 °C under aerobic conditions. Strong interactions between *H. pylori* proteins yielded red and blue colonies on MC-Mal and LB-X-Gal, respectively (see supplementary Fig. S1 and S2 for ApbC interactions, and Fig. S3 for FdxA interactions). T18-fusions with T25 (vector only) were also included as negative controls in each screening (see supplementary Fig. S4). Results obtained on both chromogenic media were in good agreement (*e.g.*, clones turning red on MC-Mal were blue on LB-X-Gal, while white clones on MC-Mal were also white on LB-X-Gal, see Fig. S1, S2 and S3). ApbC was found to interact with 19 putative Fe–S-containing proteins, including itself (HP0207), NifU (HP0221), FdxA (HP0277) and Nfu (HP1492) (Fig. S1 and S2). Other proteins strongly interacting with ApbC included the fumarate reductase subunit FrdB (HP0191), the 2-oxoglutarate oxidoreductase subunit OorD (HP0588), the pyruvate-ferredoxin oxidoreductase subunit (HP1109), as well as two components of the essential NADH:ubiquinone respiratory pathway, Nqo3 (HP1266) and Nqo9 (HP1268) (Fig S1 and S2). The hydrogenase subunit HynA (HP0631) and aconitase (HP0779) were also among the T18-protein fusions able to activate the BACTH system in presence of T25-ApbC (Fig. S1 and S2).

Likewise, FdxA was shown to interact with 12 predicted Fe–S-containing proteins, including itself (HP0277), ApbC (HP0207) and Nfu (HP1492) (Fig. S3). Furthermore, proteins such as the dual-specificity RNA methyltransferase RlmN (HP1428) and the [2Fe–2S] Ubiquinol cytochrome *c* oxidoreductase Rieske (HP1540) were found to interact with FdxA (Fig. S3).

Since neither screening medium was sensitive enough to detect weak interactions or to discriminate between weak interactions and negative controls (even after prolonged incubation times), we monitored the growth of (co-transformed) *E. coli* cells in M63-Mal minimal medium, as previously described^29^. Briefly, the duration and/or strength of T18-protein/T25-protein interactions leads to the cAMP-CRP activation of the *mal* operon, allowing *E. coli cya* mutants to use maltose and grow in the minimal medium. The growth (OD_595_), recorded after 72 h incubation at 30 °C under aerobic condition, was scored as follows: OD_595_ ≤ 0.05 (white boxes), no growth, *e.g.* no detectable interaction, including vector-only negative controls; 0.05 < OD_595_ < 0.1 (yellow boxes), weak interactions; 0.1 ≤ OD_595_ < 0.2 (blue boxes), intermediate interactions; OD_595_ ≥ 0.2 (green boxes), strong interactions (Table 2). Overall, most protein–protein interactions detected on (MC-Mal and LB-X-Gal) chromogenic media correlated with detectable growth (*e.g.* OD595 > 0.05) in the M63-Mal selection medium, although there were a few discrepancies between media; for instance we observed ApbC-NifU interactions on both MC-Mal and LB-X-Gal (Fig. S1 and S2) however cells harboring this combination failed to grow in M63-Mal liquid medium (Table 2). Nevertheless, M63-Mal growth experiments revealed additional interactions (not detected on the chromogenic screening media), for instance (i) between ApbC and three hypothetical proteins (HP0117, HP0138, HP0568), MiaB (HP0285), MoaA (HP0768) or QueA (HP0934), and (ii) between FdxA and the hydrogenase [4Fe–4S] subunit HynA (HP0631), MqnE (HP0654), MqnC (HP0656), QueA(HP0934), AddB (HP1089), NuoB (HP1261) or RlmN (HP1428).

**Table 2:**
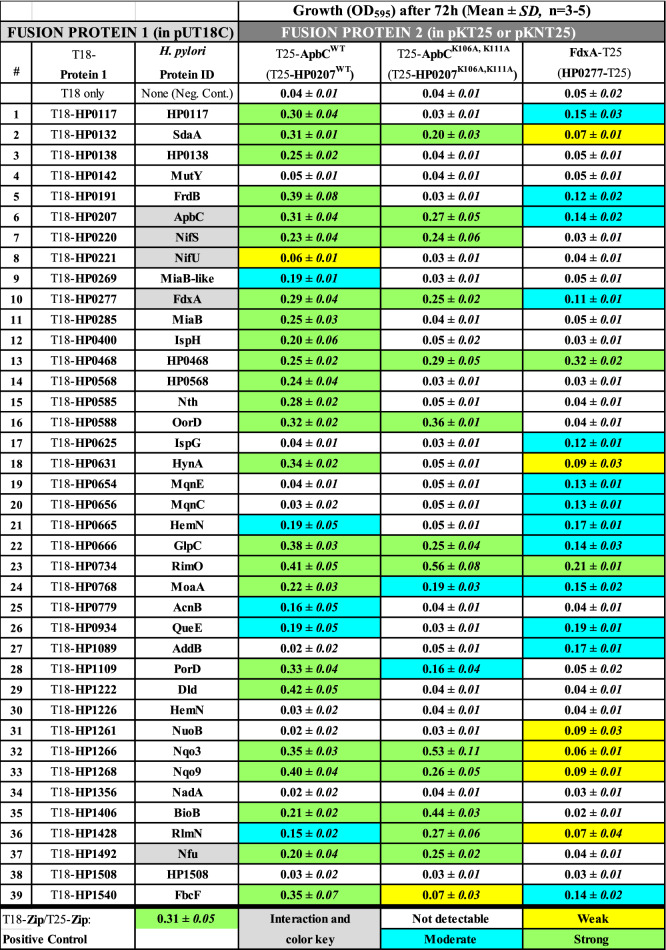
Growth of co-transformed *E. coli* BTH101 cells in M63-maltose minimal medium.

Taken together, those results suggest that ApbC is a major actor of the NIF maturation system, since the protein appears to interact with NifS (Table 2), NifU (Fig. S1 and S2) and with most (29 out of 37) of the Fe–S proteins, including itself (HP0207), FdxA and Nfu (Table 2, Fig. S1 and S2). The number of proteins interacting with FdXA was slightly more limited compared to ApbC’s network; nonetheless FdxA was found to interact with NifS and 20 putative Fe–S proteins, including itself (HP0277), ApbC and Nfu. Interestingly, no interaction between NifU and FdxA was observed, suggesting the ferredoxin-like protein FdxA might acquire its [4Fe-4S] cluster content from another source; ApbC is a good candidate, since both proteins appear to strongly interact together, as shown on both solid media (Fig. S1 and S2), as well as with the M63-Mal liquid growth assay (Table 2). Table 3 summarizes all interactions between the five proteins identified so far as core components of the NIF maturation pathway (e.g. ApbC, FdxA, Nfu, NifS, NifU), based on results herein as well as previously published data^29^.

**Table 3:** Summary of interactions between core components of the *H. pylori* NIF system.

T25 fusion	T18 fusion				
	T18-ApbC	T18-NifS	T18-NifU	T18-FdxA	T18-Nfu
T25-ApbC	(+) 3^abc^/3^abc^	(+) 1^c^/3^abc^	(+) 2^ab^/3^abc^	(+) 3^abc^/3^abc^	(+) 4^abcd^/4^abcd^
FdxA-T25	(+) 3^abc^/3^abc^	(+) 1^a^/3^abc^	(−) 0/3^abc^	(+) 3^abc^/3^abc^	(+) 1^a^/3^abc^
T25-NifS	(+) 2^fg^/3^efg^	(+) 4^efgh^/4^efgh^	(+) 4^efgh^/4^efgh^	(+) 1^g^/3^efg^	(+) 1^g^/3^efg^
T25-NifU	(+) 3^efg^/3^efg^	(+) 4^efgh^/4^efgh^	(+) 4^efgh^/4^efgh^	(−) 0/3^efg^	(+) 3^efg^/3^efg^
T25-Nfu	(+) 4^defg^/4^defg^	(+) 1^g^/3^efg^	(+) 1^h^/3^fgh^	(+) 3^efg^/3^efg^	(+) 4^efgh^/4^efgh^

( +): interactions have been detected, at least with one method; (−): no interaction could be detected. Fraction numbers indicate the numbers of methods showing interactions over the total number of methods used. Superscript letters refer to the detection method: (a) MC-Mal (this study); (b) LB-X-Gal (this study); (c) M63-Mal (this study); (d) cross-linking (this study); (e) MC-Mal^29^; (f) LB-X-Gal^29^; (g) M63-Mal^29^;(h) SEC^29^.

### A functional ATPase motif is needed for some ApbC-(Fe–S) protein interactions

Previous studies have shown that neither the presence of ATP nor the functional role of the ATPase domain is needed for the [Fe–S] cluster transfer activity of *Salmonella* ApbC^35^. However, the ATPase activity might be needed for the actual interaction of ApbC with [Fe–S] cluster donors (such as NifU) or with [Fe–S] cluster recipients (such as FdxA, or any other target Fe–S protein). Such possibility was addressed in this study, by mutagenizing the conserved ATPase motif known as the deviant Walker box (GK^106^GGVGK^111^S, see Fig. 1). Site-directed mutagenesis of the *apbC* gene was carried out, leading to the replacement of both conserved lysine residues (*e.g*., K106 and K111) by two alanine residues. The mutated gene was cloned in pKT25 and the recombinant plasmid was introduced in *E. coli* BTH101 (*cya* mutant), along with each of the previously described pUT18C fusion plasmids^29^. Protein–protein interactions involving the T25-ApbC^K106A, K111A^ variant were investigated using the same approach as that used to study T25-ApbC^WT^. Results obtained with MC-Mal and LB-X-Gal solid media are shown in Fig. S1 and S2 (alongside T25-ApbC^WT^ interaction results, to allow for direct comparison). Of interest, strong interactions between ApbC^WT^ and some Fe–S target proteins, such as HP0191 (fumarate reductase subunit) or HP0779 (aconitase), were not present anymore with the variant (Fig. S1 and S2). These decreased levels of interaction were confirmed when co-transformed BTH101 cells were grown in M63-Mal minimal medium growth experiments (Table 1). Overall, alteration of the ApbC ATPase motif resulted in a substantial decrease in protein–protein interactions: indeed, almost 50% of the interactions observed between (T25-) ApbC^WT^ and (Fe–S) target proteins were not observed with the (T25-ApbC^K106A, K111A^ variant (Table 2). These results strongly suggest that ATP binding and/or hydrolysis is important for *H. pylori* ApbC’s ability to interact with (Fe–S) target proteins.

### A heterodimeric ApbC-Nfu complex can be captured by cross-linking

**Figure 3:**
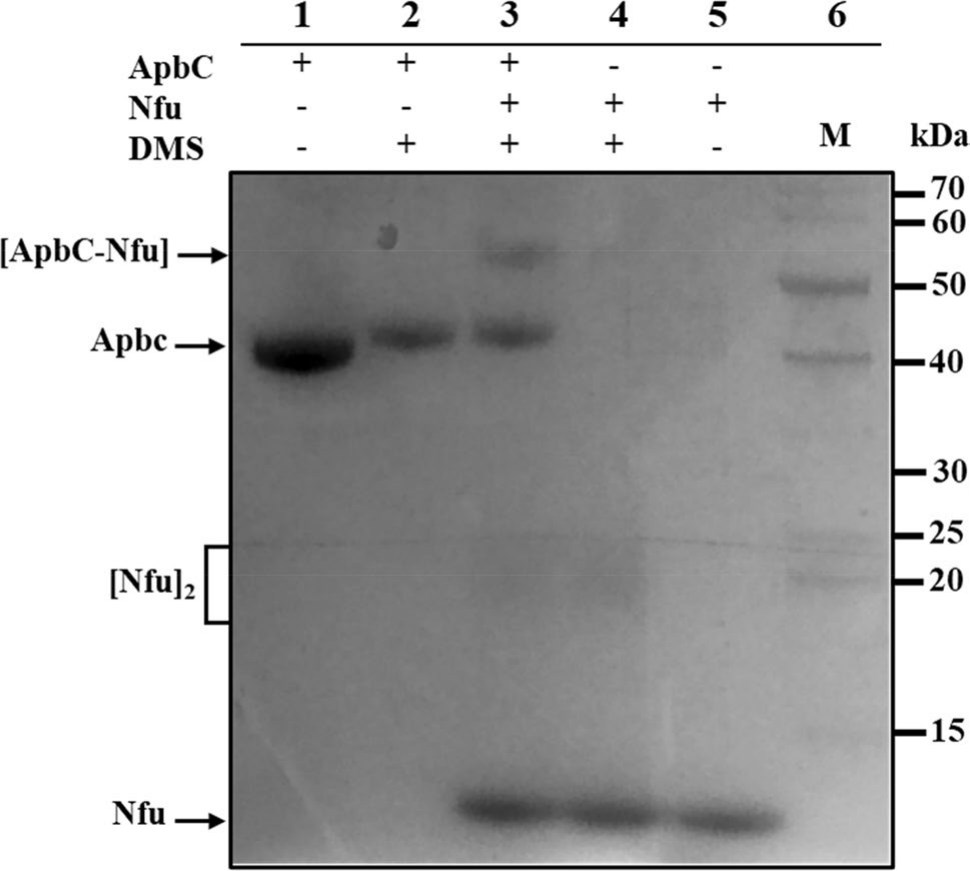
Picture of a gel showing interactions between recombinant ApbC and Nfu proteins. Purified recombinant hexahistidine tagged-ApbC^WT^ (theoretical mass: 41.1 kDa) and recombinant Nfu (10. 1 kDa) were incubated alone or together for 1 h at 25 °C, in absence or presence of the cross linker DMS. Complexes were separated on SDS 4–20% gradient PAGE and stained with Coomassie blue. Conditions are indicated above each lane. Monomeric and dimeric protein complexes are indicated by arrows on the left. A molecular weight ladder was loaded in lane 6, with corresponding molecular masses shown on the right.

Based on previously published BACTH results (using Nfu/HP1492 as bait)^29^, and results obtained herein (Table 2, Fig. S1 and S2), ApbC and Nfu are expected to interact together. To investigate this possibility, purified ApbC-(His)_6_ (predicted mass: 41.1 kDa) and Nfu (10.1 kDa) were incubated, by themselves or together, with or without addition of the homobifunctional dimethyl suberimidate (DMS) cross-linker. The protein complexes were subjected to SDS 4-20% gradient PAGE and stained with Coomassie blue (Fig. 3). Upon addition of DMS, Nfu homodimers of approximate mass 20–25 kDa can be seen (Fig. 3, lanes 3 and 4). Incubation of ApbC along with Nfu in the presence of DMS resulted in the formation of a unique adduct (Fig. 3, lane 3) with a molecular mass expected for a heterodimeric ApbC-Nfu complex (calculated mass: approximately 51.2 kDa). The complex was not seen when either of these proteins was tested individually, with or without DMS (Fig. 3, lanes 1, 2, 4 and 5). Interaction between Nfu and the ApbC^K106A, K111A^ variant was not tested in the present study, since it appears the ATPase activity is not required for such interaction. This statement is based upon the fact that (i) the cross-linking reaction was done with purified proteins in absence of ATP; (ii) Nfu interactions with the ApbC^K106A, K111A^ variant appear as strong with ApbC^WT^, as shown by BACTH (Table 2, Fig. S1 and S2).

In summary it appears that ApbC and Nfu, both of which are putative Fe–S carriers, intimately interact to form a complex, which is likely involved in Fe–S cluster(s) transfer, although this remains to be experimentally proven. Attempts to show transfer Fe–S from one (holo) protein to the other (apo) protein, using Raman resonance, were unsuccessful (data not shown).

### Proposed role for ApbC and FdXA in *H. pylori*

**Figure 4:**
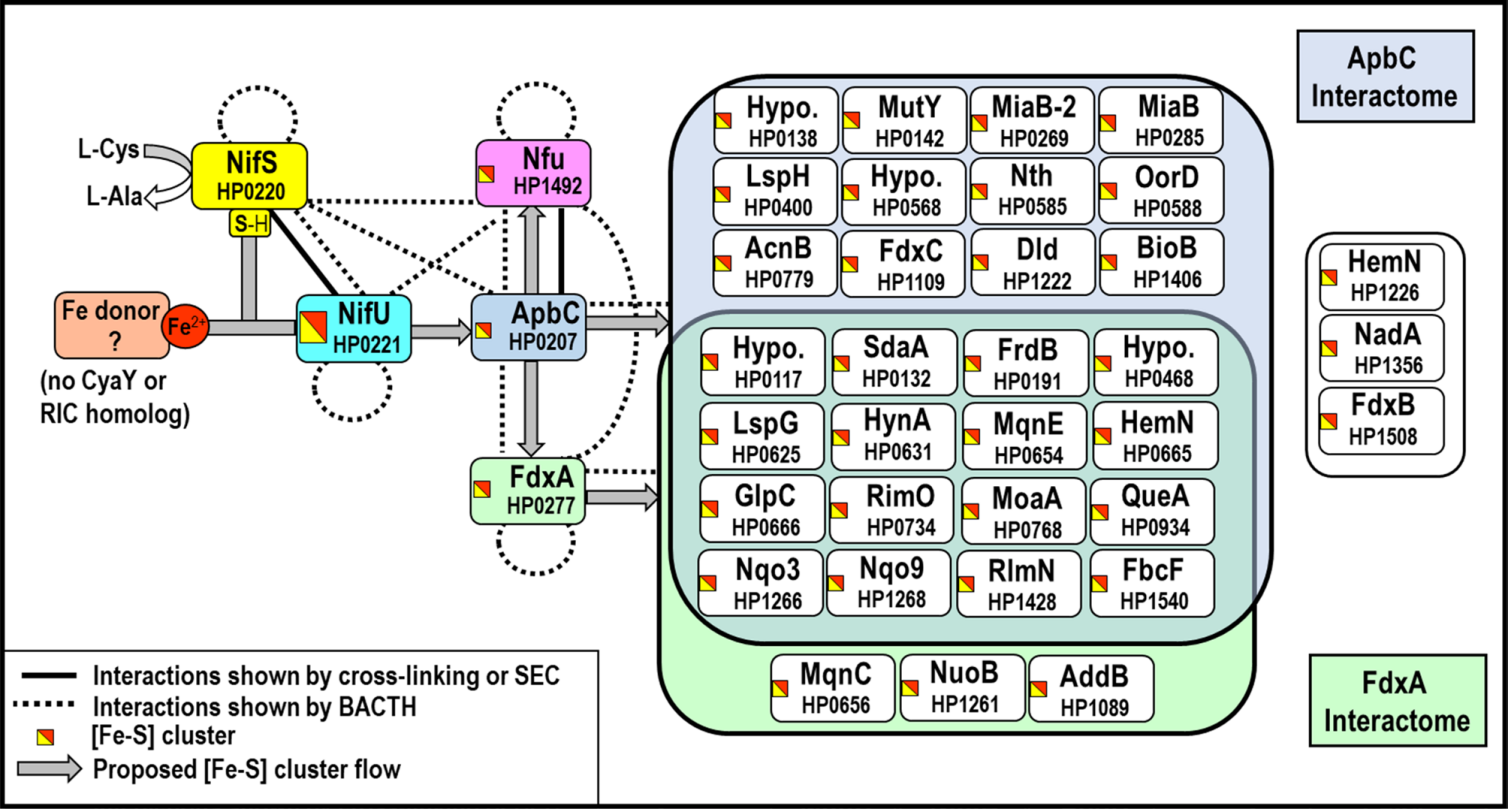
Proposed model for [Fe–S] clusters homeostasis in *H. pylori* and summary of interactions involving ApbC (HP0207) and FdxA (HP0277). Sulfur (originating from NifS) and iron (free, or brought by a protein yet to be identified) are assembled as [Fe–S] clusters on the NifU scaffold protein (or alternatively on ApbC) and subsequently distributed via three Fe–S carrier proteins, ApbC, FdxA and Nfu; and eventually to [Fe–S] cluster recipient proteins. Based on BACTH (screening and selection) results, proteins found to interact with either ApbC or FdxA are shown in respective interaction balloons. Proteins shown on the right were not found to interact with any of the bait proteins. NifS, NifU and Nfu interactomes have been previously described^29^.

The network of interactions gathered from this study, complemented by the previous one,^29^ is summarized in Fig. 4. Regarding ApbC, we propose a role in scaffolding and/or transferring Fe–S clusters, based on (i) significant homology between HP0207 and other members of the Nbp35/ApbC previously shown to coordinate and transfer [4Fe-4S] clusters, such as *S.* Typhimurium ApbC (Fig. 1) or *M. maripaludis* MMP0704; (ii) protein–protein interactions between HP0207 and numerous Fe–S target proteins, as well as NifS, as revealed by BACTH results in the present study (Table 2, Fig. S1); and (iii) interactions between ApbC and Nfu, as shown by cross-linking (Fig. 3). The possibility of ApbC being a standalone scaffold relies on the fact that ApbC appears to directly interact with the L-cysteine desulfurase NifS, as suggested by the following lines of evidence. First, using NifS as bait in our previously published BACTH study, we identified ApbC as one of the few interacting partners^29^. Second, the reverse is true: when ApbC was used as bait, as described in the present BACTH study, we identified NifS as interacting partner (Table 1). Attempts were made to reconstitute Fe–S clusters on purified recombinant ApbC, using *Azotobacter vinelandii* IscS as S-donor (an approach successfully used with HP1492/Nfu in the past^29^) however they were unsuccessful (data not shown). More work will be required to prove (or disprove) that ApbC can play a role as a standalone scaffold in the NIF pathway.

Alternatively, the possibility of an ApbC-NifS-NifU multi-component complex, on which Fe–S recipient proteins would bind to acquire their Fe–S clusters, should not be discarded. Previous results obtained in *E. coli* are in support of such hypothesis: for instance, the *E. coli* NifS homolog, IscS, has been shown to form complexes with the NifU homolog, IscU, as well as several other partners, such as ferredoxin^51^, TusA^52^, bacterial frataxin CyaY^53^ and the ancillary protein IscX^54,55^. A model involving a ternary complex IscS-IscU-CyaY has even been proposed^55^. Likewise, a FdxA-NifS-NifU multi-component platform is also a possibility in *H. pylori*, On one hand, both NifS-NifU and NifS-FdxA have been identified (based on^29^ and present study); on the other hand, NifU-FdxA interactions have not been identified (in the present study), however one has to remember that the BACTH system only allows for direct (protein–protein) interaction. Hence, a heterotrimeric complex involving FdxA, NifS and NifU (without direct interaction between NifU and FdxA) should not be ruled out.

Whether ApbC is a standalone scaffold or a Fe–S carrier protein, nonetheless it is expected to play a central role in the NIF maturation system, given that its interactome is larger than that of FdxA or Nfu (Fig. 4) and *apbC* mutants are not viable, in contrast to *fdxA* and *nfu* mutants. Since ApbC can interact with NifU and with FdxA, whereas these two latter proteins do not appear to interact with one another (Table 3, Fig. S2), this observation would support a model where Fe–S clusters flow according to the following scheme: NifU → ApbC → FdxA → (Fe–S) cluster recipient proteins (Fig. 4). More work is needed to better understand NIF-mediated [Fe–S] cluster maturation in bacteria. For instance, it would be interesting to confirm the ApbC-FdxA interaction (as suggested by BACTH results in the present study), using purified ApbC and FdxA proteins, and cross-linking. This will be the subject of future studies. The fact that NIF is the only pathway in *H. pylori* makes the gastric pathogen a unique and attractive bacterial model, hence more research on (Fe–S) cluster homeostasis will be conducted on this organism in the future.

## Experimental procedures

### Bacterial strains and plasmids

*E. coli* and *H. pylori* strains, and plasmids used in this study, are listed in Table S1. Genomic DNA from *H. pylori* strain 26695 was used as template for all PCR amplifications. All plasmids were sequenced at the Georgia Genomics Facility, University of Georgia, Athens, GA or at ETON Bioscience Inc., Research Triangle Park, NC.

### Growth conditions

*E. coli* cells were grown aerobically in Luria–Bertani (LB) broth or on LB plates at 37 ºC, unless indicated otherwise. Ampicillin (Amp, 100 µg/ml), Chloramphenicol (Cm, 25 µg/ml) and kanamycin (Kan, 30 µg/mL) were added as needed. BACTH screening media include MacConkey (MC) plates (BD-Difco # 281810), supplemented with 1% glucose-free maltose (Mal, BD-Difco #216830), or LB plates supplemented with 20 µg/mL 5-bromo-4-chloro-3-indolyl-β-d-galactopyranoside (X-Gal, Fisher). Both MC-Mal and LB-X-Gal were supplemented with 0.5 mM isopropyl β-d-1-thiogalactopyranoside (IPTG, GoldBio), 0.1 mM FeCl_3_, 0.5 mM L-cysteine, 100 µg/mL Amp and 30 µg/mL Kan. For BACTH selection experiments, we used a Fe-enriched M63 liquid minimal medium: (NH_4_)_2_SO_4_ (2 g/L), KH_2_PO_4_ (13.6 g/L), Thiamine B1 (1 mg/L), 1 mM MgSO_4_, FeSO_4_.7H_2_O (5 mg/L), pH 7. This medium was supplemented with 0.4% Mal, 0.5 mM IPTG, 50 µg/mL Amp and 15 µg/mL Kan. All BACTH growth experiments were carried out at 30 °C in aerobiosis (*e.g.* exposed to air). *H. pylori* was routinely grown on Brucella agar plates supplemented with 10% defibrinated sheep blood (BA plates), at 37 ºC under microaerobic conditions (5% CO_2_, 4% O_2_ and 91% N_2_). Cm (25 µg/ml) was added as needed.

### Construction of *H. pyloriapbC* and *fdxA* mutants

We attempted to construct *apbC* or *fdxA* deletion mutant strains by replacing either *apbC* (*hp0207*) or *fdxA* (*hp0277*) with a *cat* (chloramphenicol acetyl transferase) cassette. The strategy relies on a splicing-by-overlap-extension (SOE) polymerase chain reaction (PCR) method. It has been successfully used to construct mutants in our lab, including most recently *nfu::cat* mutants^29^. Briefly, genomic DNA from *H. pylori* WT strain 26,695 (Table S1) was used as a template for PCR to amplify fragments of DNA flanking either *hp0207* or *hp0277*^26^. Primers apbC-1 and apbC-2 (Table S2) were used to amplify a 455 bp-long DNA sequence located upstream of *apbC* and primers apbC-3 and apbC-4 were used to amplify a 410 bp-long sequence located downstream of *apbC*. Similarly, primers fdxA-1 and fdxA-2 were used to amplify a 555 bp-long DNA sequence located upstream of *fdxA* and primers fdxA-3 and fdxA-4 were used to amplify a 500 bp-long sequence located downstream of *fdxA*. Final SOE-PCR amplification steps included the two PCR products obtained for each gene deletion (e.g.* apbC* or *fdxA*), a 720 bp-long *cat* cassette^56^, and primers apbC-1 and apbC-4, or primers fdxA-1 and fdxA-4, respectively. The final PCR products (1585 bp for *apbC::cat* and 1775 bp for *fdxA::cat*, respectively) were introduced by natural transformation into various *H. pylori* parental strains (X47, 43504 and 26695) and cells were plated first on plain BA medium, and then transferred after 12 h on BA supplemented with chloramphenicol (BA-Cm)., No mutant could be recovered after transformation with the PCR product harboring the *apbC::cat* construct, despite multiple attempts. In contrast, *fdxA::cat* mutants appeared after 3 to 5 days on BA-Cm plates, but only in strain 26695^26^; no mutant could be generated in strain 43504^57^ or X47^58^. The concomitant deletion of the *hp0277* gene and the insertion of the *cat* marker in the chromosome of the 26695 *fdxA::cat* mutant were confirmed by PCR, using genomic DNA from mutants as template, and primers fdxA-1 and fdxA-4.

### Expression and purification of Nfu and ApbC

The cloning, expression and purification of Nfu has been reported^29^. ApbC was expressed as recombinant hexahistidine-tagged proteins, using *E. coli* BL21 RIL as host strain. Briefly, primers ApbC-NdeI and ApbC-XhoI (Table S2) were used to amplify a 1130 bp-long DNA sequence containing the whole native *hp0207* (*apbC*) ORF without its stop codon (using genomic DNA from *H. pylori* strain 26695 as template) as well as to incorporate a 5' *Nde*I and a 3' *Xho*I restriction site, respectively. The PCR product was digested with *Nde*I and *Xho*I, gel-purified and cloned into similarly digested pET21b plasmid, generating pET-ApbC. This plasmid was then transformed into *E. coli* BL21 RIL strain and transformants were isolated on LB supplemented with both Amp and Cm. For protein (over)expression, cells were grown at 37 °C in 800 mL of LB broth supplemented with 0.1 mM FeCl_3_, 1 mM L-cysteine, Amp (100 mg/L) and chloramphenicol (30 mg/L) to an OD_600_ of 0.3–05, cooled at 25 °C; protein expression was induced by adding 0.25 mM IPTG in the medium and leaving the cells for 3 to 4 h at 25 °C. Cells were harvested by centrifugation (15,000×*g*, 20 min, 4 °C) and subsequent steps were performed at 4 °C. Bacteria were lysed by three passages through a cold French pressure cell at 18,000 lb/in^2^, cell debris were removed by centrifugation at 15,000×*g*, and the (cell-free) supernatant was loaded onto a nickel nitrilotriacetic column (Ni–NTA). The hexahistidine-tagged ApbC^WT^ protein was purified following the manufacturer’s instructions (Qiagen, Valencia, CA). Fractions of interest were pooled, concentrated using an YM-10 cutoff Centricon device (Millipore, Billerica, MA) and subsequently subjected to stepwise buffer exchange to discard imidazole. The protein concentration was determined with the BCA protein kit (Thermo Fisher Pierce, Rockford, IL, USA).

### Crosslinking of purified ApbC and Nfu proteins

Cross-linking was done as previously reported^59^, with the following modifications. Purified recombinant native ApbC and Nfu (15 µM) were incubated for 60 min at 25 °C in the presence of 10 mM dimethyl suberimidate (DMS), a cross linker containing an amine-reactive imidoester group at each end of an 8-atom spacer arm (Thermo Fisher Pierce, Waltham, MA). The reaction was quenched by the addition of Tris-containing Laemmli denaturing loading buffer^60^. Samples were subjected to SDS-4-20% PAGE (NuSep, Bogart GA) with a Mini-Protean II apparatus (Bio-Rad, Hercules, CA), and the gel was stained with Coomassie.

### Site-directed mutagenesis of the ApbC ATPase motif

SOE-PCR was used to substitute the two conserved lysine residues (K106 and K111) for alanine residues in the ApbC ATPase motif, as follows. Briefly, genomic DNA from strain 26695 and primers ApbC-XbaI and ApbC-mut1 (Supplementary Table S2) were used to amplify a 500 bp-long DNA sequence containing the *apbC* sequence upstream of the Walker box, as well as to incorporate a *Xba*I restriction site (on the 5’ end) and both (K → A) mutations (on the 3’ end). Likewise, primers ApbC-KpnI and ApbC-mut2 were used to amplify a 825 bp-long DNA sequence containing the *apbC* sequence downstream of the Walker box, as well as to incorporate both (K → A) mutations (on the 5’ end) and a *Kpn*I restriction site (on the 3’ end). Finally, both PCR products (with overlapping sequences) were combined, along with primers ApbC-XbaI and ApbC-KpnI, to amplify a 1260 bp-long DNA sequence containing the whole (mutated) *apbC* sequence. This PCR product was digested with *Xba*I and *Kpn*I, gel-purified and cloned into similarly digested pKT25, pKNT25, or pUT18C plasmid, for further use with the BACTH system.

### Bacterial adenylate cyclase two hybrid (BACTH)

A BACTH-based kit (Euromedex, France)^33^ was used to study protein–protein interactions between ApbC^WT^, or ApbC^K106A K111A^, or FdxA, and NifS, or 38 Fe–S target proteins (including NifU and Nfu), as previously described^29^. Using primers designed to introduce a *Xba*I restriction site on the 5’ end and a *Kpn*I restriction site on the 3’ end, respectively (Table S2), a 1130 bp-long DNA sequence containing the *apbC*/*hp0207* (WT or K106A, K111A mutant) ORF and a 250 bp-long DNA sequence containing the *fdxA*/*hp0277* ORF (without start and stop codons) were PCR-amplified, digested with *Xba*I and *Kpn*I and ligated into similarly digested pKT25, pKNT25 or pUT18C plasmid to generate in-frame gene fusions (Table S1). All other genes described in this study have been previously cloned into pUT18C plasmid (Table S1)^29^. Ligation mixtures were introduced into *E. coli* TOP10 and transformants were selected on LB plates supplemented with 100 µg/mL Amp (for pUT18C derivatives) or LB plates supplemented with 30 µg/mL Kan (for pKT25 or pKNT25 derivatives). Recombinant plasmids were verified by restriction profiles and DNA sequencing. Finally, *E. coli* BTH101 (*cya* mutant, Euromedex) cells were co-transformed with a combination of one pUT18C derivative and one pK(N)T25 derivative; co-transformants were selected on LB plates supplemented with both Kan and Amp. Individual colonies were picked and grown overnight at 30 °C in LB supplemented with both antibiotics. This cell suspension was used as inoculum for all subsequent screening and growth experiments. Interactions between Cya T18- and Cya T25-fusion proteins were analyzed using three complementary methods: (1) screening on MC-Mal plates: clones positive for protein–protein interaction turned red; (2) screening on LB-X-Gal plates: clones positive for protein–protein interaction turned blue; and (3) selection in M63-Mal liquid minimal medium: protein–protein interaction is required for *E. coli cya* mutants to grow in this medium. Three negative controls were included in each experiment: (1) pUT18C-pKT25 vector only controls, (2) pUT18C vector only with pK(N)T25-gene fusion, and (3) pUT18C-gene fusions with pKNT25 vector only. A positive control (pUT18C-zip with pKT25-zip, provided with the kit) was also included in each experiment. For MC-Mal or LB-X-Gal plates, 1 µL of LB-grown cells was spotted, and plates were incubated at 30 °C for 36–48 h under aerobic conditions. For 96-well plates, 2 µL of LB-grown cells were used to inoculate 200 µL of M63 minimal medium (1:100) and plates were incubated at 30 °C for 72 h, after which A_595_ was recorded (Biotek Synergy Mx, Winooski, VT). Each plate included a blank (2 µL of LB as inoculum). Results shown are the average and SD of blank-subtracted A_595_ from 3 to 5 independent growth experiments.

### H_2_-uptake hydrogenase assays

The H_2_-uptake hydrogenase activity of wild-type strain 26695 and 26695 *fdxA* mutant was measured on whole cells by using a modified methylene blue (MB)-coupled spectrophotometric method^50^. Briefly, *H. pylori* cells were grown on BA plates under a H_2_-enriched microaerobic atmosphere^61^, harvested and suspended in H_2_-sparged phosphate-buffered saline (PBS) to a final concentration of 5 × 10^8^ to 1 × 10^9^ cells per ml. H_2_-flushed PBS and methylene blue (400 µM) were mixed with sodium dithionite (200 µM, used to scavenge any residual oxygen) in a 1.8 mL sealed glass cuvette previously flushed with H_2._. Once the baseline was stable (OD_570_ ~ 1), whole cells aliquots (100–200 µL) were added to the mixture, and H_2_ oxidation was followed by measuring the reduction (decrease in OD) of oxidized MB at 570 nm. One mole of oxidized H_2_ corresponds to 2 mol of reduced MB. Hydrogenase activity is expressed as nmoles H_2_ oxidized per min per 10^9^ cells. Hydrogenase assays were done in triplicate.

## Supplementary Information

1. Supplementary Information

2. Supplementary Information

3. Supplementary Information

4. Supplementary Information

5. Supplementary Information

## References

[1] Johnson DC, Dean DR, Smith AD, Johnson MK (2005). Structure, function, and formation of biological iron-sulfur clusters. Annu. Rev. Biochem..

[2] Lill R (2009). Function and biogenesis of iron–sulphur proteins. Nature.

[3] Rouault TA (2012). Biogenesis of iron-sulfur clusters in mammalian cells: new insights and relevance to human disease. Dis. Model Mech..

[4] Baussier, C. *et al.* Making iron-sulfur cluster: structure, regulation and evolution of the bacterial ISC system. *Advances in Microbial Physiology* Vol. 76 (Poole, R.K. ed.) 1–39 (Academic Press, 2020). 10.1016/bs.ampbs.2020.01.001 32408945

[5] Loiseau L (2005). Analysis of the heteromeric CsdA-CsdE cysteine desulfurase, assisting Fe–S cluster biogenesis in *Escherichia coli*. J. Biol. Chem..

[6] Zheng L, Cash VL, Flint DH, Dean DR (1998). Assembly of iron-sulfur clusters identification of an *iscSUA-hscBA-fd*x gene cluster from *Azotobacter vinelandii*. J. Biol. Chem..

[7] Zheng L, White RH, Cash VL, Jack RF, Dean DR (1993). Cysteine desulfurase activity indicates a role for NIFS in metallocluster biosynthesis. Proc. Natl. Acad. Sci..

[8] Outten FW, Wood MJ, Muñoz FM, Storz G (2003). The SufE protein and the SufBCD complex enhance SufS cysteine desulfurase activity as part of a sulfur transfer pathway for Fe–S cluster assembly in *Escherichia coli*. J. Biol. Chem..

[9] Loiseau L, Ollagnier-de-Choudens S, Nachin L, Fontecave M, Barras F (2003). Biogenesis of Fe–S cluster by the bacterial Suf system: sufS and sufE form a new type of cysteine desulfurase. J. Biol. Chem..

[10] Blahut, M., Sanchez, E., Fisher, C. E. & Outten, F. W. Fe–S cluster biogenesis by the bacterial Suf pathway. *Biochim. Biophys. Acta (BBA)-Mol. Cell Res.* 118829 (2020). 10.1016/j.bbamcr.2020.118829 PMC7510350 32822728

[11] Agar JN (2000). IscU as a scaffold for iron−sulfur cluster biosynthesis: Sequential assembly of [2Fe-2S] and [4Fe-4S] clusters in IscU. Biochemistry.

[12] Smith AD (2005). NifS-mediated assembly of [4Fe−4S] clusters in the N-and C-terminal domains of the NifU scaffold protein. Biochemistry.

[13] Fu W, Jack RF, Morgan TV, Dean DR, Johnson MK (1994). *nifU* gene product from *Azotobacter vinelandii* is a homodimer that contains two identical [2Fe-2S] clusters. Biochemistry.

[14] Ollagnier-de-Choudens S, Mattioli T, Takahashi Y, Fontecave M (2001). Iron-sulfur cluster assembly: characterization of IscA and evidence for a specific and functional complex with ferredoxin. J. Biol. Chem..

[15] Mapolelo DT, Zhang B, Naik SG, Huynh BH, Johnson MK (2012). Spectroscopic and functional characterization of iron-sulfur cluster-bound forms of *Azotobacter vinelandii* (^Nif^)IscA. Biochemistry.

[16] Layer, G., Ollagnier-de Choudens, S., Sanakis, Y. & Fontecave, M. Iron-sulfur cluster biosynthesis: Characterization of *Escherichia coli* CyaY as an iron donor for the assembly of [2Fe-2S] clusters in the scaffold IscU. *J. Biol. Chem.* **281**, 16256–16263. 10.1074/jbc.M513569200 (2006). 10.1074/jbc.M513569200 16603772

[17] Justino MC, Almeida CC, Goncalves VL, Teixeira M, Saraiva LM (2006). *Escherichia coli* YtfE is a di-iron protein with an important function in assembly of iron-sulphur clusters. FEMS Microbiol. Lett..

[18] Vinella D, Brochier-Armanet C, Loiseau L, Talla E, Barras F (2009). Iron-sulfur (Fe/S) protein biogenesis: Phylogenomic and genetic studies of A-type carriers. PLoS Genet.

[19] Loiseau L (2007). ErpA, an iron–sulfur (Fe–S) protein of the A-type essential for respiratory metabolism in *Escherichia coli*. Proc. Natl. Acad. Sci..

[20] Iwema T (2009). Structural basis for delivery of the intact [Fe2S2] cluster by monothiol glutaredoxin. Biochemistry.

[21] Bandyopadhyay S (2008). Chloroplast monothiol glutaredoxins as scaffold proteins for the assembly and delivery of [2Fe-2S] clusters. EMBO J..

[22] Shakamuri P, Zhang B, Johnson MK (2012). Monothiol glutaredoxins function in storing and transporting [Fe2S2] clusters assembled on IscU scaffold proteins. J. Am. Chem. Soc..

[23] Jacobson MR (1989). Biochemical and genetic analysis of the nifUSVWZM cluster from *Azotobacter vinelandii*. Mol. Gen. Genet. MGG.

[24] Gao H (2013). *Arabidopsis thaliana* Nfu2 accommodates [2Fe-2S] or [4Fe-4S] clusters and is competent for in vitro maturation of chloroplast [2Fe-2S] and [4Fe-4S] cluster-containing proteins. Biochemistry.

[25] Yabe T (2008). Structural analysis of *Arabidopsis* CnfU protein: An iron-sulfur cluster biosynthetic scaffold in chloroplasts. J. Mol. Biol..

[26] Tomb JF (1997). The complete genome sequence of the gastric pathogen *Helicobacter pylori*. Nature.

[27] Tokumoto U, Kitamura S, Fukuyama K, Takahashi Y (2004). Interchangeability and distinct properties of bacterial Fe–S cluster assembly systems: functional replacement of the isc and suf operons in *Escherichia coli* with the nifSU-like operon from *Helicobacter pylori*. J. Biochem..

[28] Olson JW, Agar JN, Johnson MK, Maier RJ (2000). Characterization of the NifU and NifS Fe–S cluster formation proteins essential for viability in *Helicobacter pylori*. Biochemistry.

[29] Benoit SL, Holland AA, Johnson MK, Maier RJ (2018). Iron–sulfur protein maturation in *Helicobacter pylori:* Identifying a Nfu-type cluster carrier protein and its iron–sulfur protein targets. Mol. Microbiol..

[30] Chiou, P.-Y., Luo, C.-H., Chang, K.-C. & Lin, N.-T. Maintenance of the cell morphology by MinC in *Helicobacter pylori*. *PLoS One* **8** (2013). 10.1371/journal.pone.0071208 PMC3731275 23936493

[31] Benoit S, Maier RJ (2003). Dependence of *Helicobacter pylori* urease activity on the nickel-sequestering ability of the UreE accessory protein. J. Bacteriol..

[32] Pereira L, Hoover TR (2005). Stable accumulation of sigma54 in *Helicobacter pylori* requires the novel protein HP0958. J. Bacteriol..

[33] Karimova G, Pidoux J, Ullmann A, Ladant D (1998). A bacterial two-hybrid system based on a reconstituted signal transduction pathway. Proc. Natl. Acad. Sci. U S A.

[34] Mukhopadhyay AK, Jeong J-Y, Dailidiene D, Hoffman PS, Berg DE (2003). The *fdxA* ferredoxin gene can down-regulate *frxA* nitroreductase gene expression and is essential in many strains of *Helicobacter pylori*. J. Bacteriol..

[35] Boyd JM, Pierik AJ, Netz DJ, Lill R, Downs DM (2008). Bacterial ApbC can bind and effectively transfer iron-sulfur clusters. Biochemistry.

[36] Skovran E, Downs DM (2003). Lack of the ApbC or ApbE protein results in a defect in Fe–S cluster metabolism in *Salmonella enterica* serovar Typhimurium. J. Bacteriol..

[37] Boyd JM, Drevland RM, Downs DM, Graham DE (2009). Archaeal ApbC/Nbp35 homologs function as iron-sulfur cluster carrier proteins. J. Bacteriol..

[38] Zhao C (2019). The Nbp35/ApbC homolog acts as a nonessential [4Fe-4S] transfer protein in methanogenic archaea. FEBS Lett..

[39] Hausmann A (2005). The eukaryotic P loop NTPase Nbp35: an essential component of the cytosolic and nuclear iron–sulfur protein assembly machinery. Proc. Natl. Acad. Sci..

[40] Roy A, Solodovnikova N, Nicholson T, Antholine W, Walden WE (2003). A novel eukaryotic factor for cytosolic Fe–S cluster assembly. EMBO J..

[41] Pardoux R (2019). The bacterial MrpORP is a novel Mrp/NBP35 protein involved in iron-sulfur biogenesis. Sci. Rep..

[42] Netz DJ, Pierik AJ, Stümpfig M, Mühlenhoff U, Lill R (2007). The Cfd1–Nbp35 complex acts as a scaffold for iron-sulfur protein assembly in the yeast cytosol. Nat. Chem. Biol..

[43] Netz DJA (2012). A bridging [4Fe-4S] cluster and nucleotide binding are essential for function of the Cfd1-Nbp35 complex as a scaffold in iron-sulfur protein maturation. J. Biol. Chem..

[44] Benoit, S. L. & Maier, R. J. Twin-arginine translocation system in *Helicobacter pylori*: TatC, but not TatB, is essential for viability. *mBio* 5, e01016–01013, 10.1128/mBio.01016-13 (2014). 10.1128/mBio.01016-13 PMC3903283 24449753

[45] Petersen L, Downs DM (1996). Mutations in *apbC* (*mrp*) prevent function of the alternative pyrimidine biosynthetic pathway in *Salmonella typhimurium*. J. Bacteriol..

[46] Boyd JM, Lewis JA, Escalante-Semerena JC, Downs DM (2008). *Salmonella enterica* requires ApbC function for growth on tricarballylate: Evidence of functional redundancy between ApbC and IscU. J. Bacteriol..

[47] Jaroschinsky M, Sawers RG (2014). Ferredoxin has a pivotal role in the biosynthesis of the hydrogen-oxidizing hydrogenases in *Escherichia coli*. Int. J. Hydrogen Energy.

[48] van Vliet AH, Baillon M-LA, Penn CW, Ketley JM (2001). The iron-induced ferredoxin FdxA of *Campylobacter jejuni* is involved in aerotolerance. FEMS Microbiol. Lett..

[49] Benoit, S. L., Maier, R. J., Sawers, R. G. & Greening, C. Molecular hydrogen metabolism: a widespread trait of pathogenic bacteria and protists. *Microbiol. Mol. Biol. Rev.* **84**, 10.1128/MMBR.00092-19 (2020). 10.1128/MMBR.00092-19 PMC7167206 31996394

[50] Lamichhane-Khadka, R., Kwiatkowski, A. & Maier, R. J. The Hyb hydrogenase permits hydrogen-dependent respiratory growth of *Salmonella enterica* serovar Typhimurium. *mBio* **1**, 10.1128/mBio.00284-10 (2010). 10.1128/mBio.00284-10 PMC3000549 21157514

[51] Yan R (2013). Ferredoxin competes with bacterial frataxin in binding to the desulfurase IscS. J. Biol. Chem..

[52] Shi R (2010). Structural basis for Fe–S cluster assembly and tRNA thiolation mediated by IscS protein-protein interactions. PLoS Biol..

[53] Prischi F (2010). Structural bases for the interaction of frataxin with the central components of iron-sulphur cluster assembly. Nat. Commun..

[54] Kim JH, Bothe JR, Frederick RO, Holder JC, Markley JL (2014). Role of IscX in iron-sulfur cluster biogenesis in *Escherichia coli*. J. Am. Chem. Soc..

[55] di Maio D, Chandramouli B, Yan R, Brancato G, Pastore A (1861). Understanding the role of dynamics in the iron sulfur cluster molecular machine. Biochem. Biophys. Acta..

[56] Wang Y, Taylor DE (1990). Chloramphenicol resistance in *Campylobacter coli:* Nucleotide sequence, expression, and cloning vector construction. Gene.

[57] Kinoshita-Daitoku, R. *et al.* Complete genome sequence of *Helicobacter pylori* strain ATCC 43504, a type strain that can infect gerbils. *Microbiol. Resour. Announc.* **9**, 10.1128/MRA.00105-20 (2020). 10.1128/MRA.00105-20 PMC7193922 32354967

[58] Veyrier, F. J., Ecobichon, C. & Boneca, I. G. Draft genome sequence of strain X47–2AL, a feline *Helicobacter pylori* isolate. *Genome Announc.* **1**, 10.1128/genomeA.01095-13 (2013). 10.1128/genomeA.01095-13 PMC3868871 24356847

[59] Benoit, S. L., Mehta, N., Weinberg, M. V., Maier, C. & Maier, R. J. Interaction between the *Helicobacter pylori* accessory proteins HypA and UreE is needed for urease maturation. *Microbiology (Reading, England)* **153**, 1474 (2007). 10.1099/mic.0.2006/003228-0 PMC2553680 17464061

[60] Laemmli UK (1970). Cleavage of structural proteins during the assembly of the head of bacteriophage T4. Nature.

[61] Maier RJ, Olczak A, Maier S, Soni S, Gunn J (2004). Respiratory hydrogen use by *Salmonella enterica* serovar Typhimurium is essential for virulence. Infect. Immun..

