# Supplementary material for: A two-hybrid system reveals previously uncharacterized protein–protein interactions within the Helicobacter pylori NIF iron–sulfur maturation system
Source: Sci Rep. 2021 May 24;11:10794. doi: 10.1038/s41598-021-90003-1 (PMC8144621; doi:10.1038/s41598-021-90003-1)
Supplement: Supplementary file 1 — 1. Supplementary Information [file 41598_2021_90003_MOESM1_ESM.pdf]

Supplementary Tables S1 and S2 for:

"A two-hybrid system reveals previously uncharacterized protein-protein interactions within the *Helicobacter pylori* NIF iron-sulfur maturation system".

Benoit, Agudelo, Maier, *Scientific Reports*, 2021.

Table S1. Strains and plasmids used in this study

Strain or plasmid	Relevant characteristics	Source or reference
**Strain**		
E. coli		
TOP10	Cloning strain	Invitrogen
Rosetta (DE3) RIL	BL21 derivative. Host for protein overproduction; Cm^r	Novagen
BTH101	*cya-99* mutant. Host for BACTH system	(1)
H. pylori		
26695	Parental strain	(2)
26695 Δ*fdxA*	26695 with Δ*fdxA::cat*	This study
43504	Parental strain	(3)
X-47	Parental strain, mouse colonizing	(4)
**Plasmid**		
pET21b	Cloning and expression vector; Amp^r	Invitrogen
pET-$ApbC^{WT}$	pET21b with *hp0207* ORF cloned at *Nde*I-*Xho*I; Amp^r	This study
pET-Nfu	pET21b with *hp1492* ORF cloned at *Nde*I-*Xho*I; Amp^r	(5)
pUC20-cat	Source of *cat* cassette; Cm^r	(6)
pKT25	BACTH; allows for C-terminal fusions to T25 fragment of CyaA; Kan^r	(1)
pKNT25	BACTH; allows for N-terminal fusions to T25 fragment of CyaA; Kan^r	(1)
pUT18	BACTH; allows for N-terminal fusions to T18 fragment of CyaA; Amp^r	(1)
pUT18C	BACTH; allows for C-terminal fusions to T18 fragment of CyaA; Amp^r	(1)
pKT25-zip	pKT25 with zip gene; positive control for BACTH; Kan^r	(1)
pUT18C-zip	pUT18C with zip gene; positive control for BACTH; Amp^r	(1)
pKT25-$ApbC^{WT}$	pKT25 with *hp0207* ORF cloned at *Xba*I- *Kpn*I; Kan^r	This study
pKNT25-$ApbC^{WT}$	pKNT25 with *hp0207* ORF cloned at *Xba*I- *Kpn*I; Kan^r	This study
pUT18C-$ApbC^{WT}$	pUT18C with *hp0207* ORF cloned at *Xba*I- *Kpn*I; Amp^r	(5)
pKT25-$ApbC^{K106A,K111A}$	pKT25 with *hp0207* (K106A, K111A) ORF cloned at *Xba*I- *Kpn*I; Kan^r	This study
pKNT25-$ApbC^{K106A,K111A}$	pKNT25 with *hp0207* (K106A, K111A) ORF cloned at *Xba*I- *Kpn*I; Kan^r	This study
pUT18C-$ApbC^{K106A,K111A}$	pUT18C with *hp0207* (K106A, K111A) ORF cloned at *Xba*I- *Kpn*I; Amp^r	This study
pKT25-FdxA	pKT25 with *hp0277* ORF cloned at *Xba*I- *Kpn*I; Kan^r	This study

pKNT25-FdxA	pKNT25 with *hp0277* ORF cloned at *Xba*I- *Kpn*I; Kan^r	This study
pUT18C-FdxA	pUT18C with *hp0277* ORF cloned at *Xba*I- *Kpn*I; Amp^r	(5)
pUT18C-HP0117	pUT18C with *hp0117* (hypothetical) ORF cloned at *Pst*I- *Kpn*I; Amp^r	(5)
pUT18C-HP0132	pUT18C with *hp0132* (*sdaA*) ORF cloned at *Xba*I- *Kpn*I; Amp^r	(5)
pUT18C-HP0138	pUT18C with *hp0138* (hypothetical) ORF cloned at *Xba*I- *Kpn*I; Amp^r	(5)
pUT18C-HP0142	pUT18C with *hp0142* (*mutY*) ORF cloned at *Xba*I- *Kpn*I; Amp^r	(5)
pUT18C-HP0191	pUT18C with *hp0191* (*frdA*) ORF cloned at *Xba*I- *Kpn*I; Amp^r	(5)
pUT18C-HP0220	pUT18C with *hp0220* (*nifS*) ORF cloned at *Xba*I- *Kpn*I; Amp^r	(5)
pUT18C-HP0221	pUT18C with *hp0221* (*nifU*) ORF cloned at *Xba*I- *Kpn*I; Amp^r	(5)
pUT18C-HP0269	pUT18C with *hp0269* (*miaB-like*) ORF cloned at *Xba*I- *Kpn*I; Amp^r	(5)
pUT18C-HP0285	pUT18C with *hp0285* (*miaB*) ORF cloned at *Xba*I- *Kpn*I; Amp^r	(5)
pUT18C-HP0400	pUT18C with *hp0400* (*ispH*) ORF cloned at *Xba*I- *Kpn*I; Amp^r	(5)
pUT18C-HP0468	pUT18C with *hp0468* (hypothetical) ORF cloned at *Xba*I- *Kpn*I; Amp^r	(5)
pUT18C-HP0568	pUT18C with *hp0568* (hypothetical) ORF cloned at *Xba*I- *Kpn*I; Amp^r	(5)
pUT18C-HP0585	pUT18C with *hp0585* (*nth*) ORF cloned at *Xba*I- *Kpn*I; Amp^r	(5)
pUT18C-HP0588	pUT18C with *hp0588* (*oorD*) ORF cloned at *Xba*I- *Kpn*I; Amp^r	(5)
pUT18C-HP0625	pUT18C with *hp0625*(*ispG*) ORF cloned at *Xba*I- *Kpn*I; Amp^r	(5)
pUT18C-HP0631	pUT18C with *hp0631* (*hynA*) ORF cloned at *Xba*I- *Kpn*I; Amp^r	(5)
pUT18C-HP0654	pUT18C with *hp0654* (*mqnD*) ORF cloned at *Xba*I- *Kpn*I; Amp^r	(5)
pUT18C-HP0656	pUT18C with *hp0656* (*mqnC*) ORF cloned at *Xba*I- *Kpn*I; Amp^r	(5)
pUT18C-HP0665	pUT18C with *hp0665* (*hemN*) ORF cloned at *Xba*I- *Kpn*I; Amp^r	(5)
pUT18C-HP0666	pUT18C with *hp0666* (*glpC*) ORF cloned at *Xba*I- *Kpn*I; Amp^r	(5)
pUT18C-HP0734	pUT18C with *hp0734* (*rimO*) ORF cloned at *Xba*I- *Kpn*I; Amp^r	(5)
pUT18C-HP0768	pUT18C with *hp0768* (*moaA*) ORF cloned at *Xba*I- *Kpn*I; Amp^r	(5)
pUT18C-HP0779	pUT18C with *hp0779* (*acnB*) ORF cloned at *Xba*I- *Kpn*I; Amp^r	(5)
pUT18C-HP0934	pUT18C with *hp0934* (*queE*) ORF cloned at *Xba*I- *Kpn*I; Amp^r	(5)
pUT18C-HP1089	pUT18C with *hp1089* (*addB*) ORF cloned at *Xba*I- *Kpn*I; Amp^r	(5)
pUT18C-HP1109	pUT18C with *hp1109* (*porD*) ORF cloned at *Xba*I- *Kpn*I; Amp^r	(5)
pUT18C-hp1222	pUT18C with *hp1222* (*dld*) ORF cloned at *Xba*I- *Kpn*I; Amp^r	(5)
pUT18C-HP1226	pUT18C with *hp1226* (*hemN*) ORF cloned at *Xba*I- *Kpn*I; Amp^r	(5)
pUT18C-HP1261	pUT18C with *hp1261* (*nuoB*) ORF cloned at *Xba*I- *Kpn*I; Amp^r	(5)
pUT18C-HP1266	pUT18C with *hp1266* (*nqoB*) ORF cloned at *Xba*I- *Kpn*I; Amp^r	(5)
pUT18C-HP1268	pUT18C with *hp1268* (*nuoI*) ORF cloned at *Xba*I- *Kpn*I; Amp^r	(5)
pUT18C-HP1356	pUT18C with *hp1356* (*nadA*) ORF cloned at *Xba*I- *Kpn*I; Amp^r	(5)
pUT18C-HP1406	pUT18C with *hp1406* (*bioB*) ORF cloned at *Xba*I- *Kpn*I; Amp^r	(5)
pUT18C-HP1428	pUT18C with *hp1428* (*rlmN*) ORF cloned at XbaI- KpnI; Amp^r	(5)
pUT18C-HP1492	pUT18C with *hp1492* (*nfu*) ORF cloned at *Xba*I- *Kpn*I; Amp^r	(5)
pUT18C-HP1508	pUT18C with *hp1508* (*ferredoxin*) ORF cloned at XbaI- KpnI; Amp^r	(5)
pUT18C-HP1540	pUT18C with *hp1540* (*fbcF*) ORF cloned at XbaI- KpnI; Amp^r	(5)

REFERENCES

1. Karimova G, Pidoux J, Ullmann A, Ladant D. 1998. A bacterial two-hybrid system based on a reconstituted signal transduction pathway. Proc Natl Acad Sci U S A 95:5752-6.
2. Tomb JF, White O, Kerlavage AR, Clayton RA, Sutton GG, Fleischmann RD, Ketchum KA, Klenk HP, Gill S, Dougherty BA, Nelson K, Quackenbush J, Zhou L, Kirkness EF, Peterson S, Loftus B, Richardson D, Dodson R, Khalak HG, Glodek A, McKenney K, Fitzegerald LM, Lee N, Adams MD, Hickey EK, Berg DE, Gocayne JD, Utterback TR, Peterson JD, Kelley JM, Cotton MD, Weidman JM, Fujii C, Bowman C, Watthey L, Wallin E, Hayes WS, Borodovsky M, Karp PD, Smith HO, Fraser CM, Venter JC. 1997. The complete genome sequence of the gastric pathogen *Helicobacter pylori*. Nature 388:539-47.
3. Kinoshita-Daitoku R, Ogura Y, Kiga K, Maruyama F, Kondo T, Nakagawa I, Hayashi T, Mimuro H. 2020. Complete genome sequence of *Helicobacter pylori* strain ATCC 43504, a type strain that can infect gerbils. Microbiol Resour Announc 9.
4. Veyrier FJ, Ecobichon C, Boneca IG. 2013. Draft genome sequence of strain X47-2AL, a feline *Helicobacter pylori* isolate. Genome Announc 1.
5. Benoit SL, Holland AA, Johnson MK, Maier RJ. 2018. Iron-sulfur protein maturation in *Helicobacter pylori*: identifying a Nfu-type cluster carrier protein and its iron-sulfur protein targets. Mol Microbiol 108:379-396.
6. Wang Y, Taylor DE. 1990. Chloramphenicol resistance in *Campylobacter coli:* nucleotide sequence, expression, and cloning vector construction. Gene 94:23-8.

Table S2. Primers used in this study

Name	Sequence (5'-3')*	Usage
ΔapbC-1	AAAGGATAGCTGATCAGCG	Construction of Δ*apbC::cat* mutant
ΔapbC-2	atccacttttcaatctatatcTAATCCTTAAAATGATAG	Construction of Δ*apbC::cat* mutant
ΔapbC-3	cccagtttgtcgcactgataaCCATGCTTGCTCGCATTAG	Construction of Δ*apbC::cat* mutant
ΔapbC-4	GATACGCTCTATTCCCTAG	Construction of Δ*apbC::cat* mutant
ΔfdxA-1	TAGCCTTAAAAGAGGGCATGC	Construction of Δ*fdxA::cat* mutant
ΔfdxA-2	atccacttttcaatctatatcGTTTGAATACGACTTTTACTC	Construction of Δ*fdxA::cat* mutant
ΔfdxA-3	cccagtttgtcgcactgataaAAGAGCAAGATTAAAGGCTAG	Construction of Δ*fdxA::cat* mutant
ΔfdxA-4	CAACATCAAGCGAGATTAAGTCC	Construction of Δ*fdxA::cat* mutant
ApbC-Xba	agtac**tctaga**gCTCACCCAAGAAGATGTC	BACTH fusion, ApbC^{WT} or ApbC^{K106A,K111A}
ApbC-Kpn	agcac**ggtacc**gaATGCGAGCAAGCATGGGT	BACTH fusion, ApbC^{WT} or ApbC^{K106A,K111A}
ApbC-NdeI	acggccc**catATG**CTCACCCAAGAAGATGTC	Expression of ApbC^{WT}
ApbC-XhoI	gcgaca**ctcgag**ATGCGAGCAAGCATGGGT	Expression of ApbC^{WT}
ApbC-mut1	GATGATAAGCTCGGGT**gcg**GGCGGTGTGGGT**gcg**AGCACCACTAGCGTG	ApbC^{K106A,K111A} mutation
ApbC-mut2	CACGCTAGTGGTGCT**cgc**AC CCACACCGCC**cgc**ACCCGAG CTTATCATC	ApbC^{K106A,K111A} mutation
FdxA-Xba	agtac**tctaga**gTCATTATTGGTGAATGATG	BACTH fusion, FdxA fusion
FdxA-Kpn	agcac**ggtacc**gaATCTTGCTCTTTTAAGC	BACTH fusion, FdxA fusion

* Upper case letters indicate *H. pylori*-derived sequences; bold letters indicate newly generated restriction sites or engineered mutations; *cat*-specific sequences used to construct mutant are underlined.
All primers were purchased from Integrated DNA Technology (IDT), Coralville, IA.
