# Supplementary material for: A two-hybrid system reveals previously uncharacterized protein–protein interactions within the Helicobacter pylori NIF iron–sulfur maturation system
Source: Sci Rep. 2021 May 24;11:10794. doi: 10.1038/s41598-021-90003-1 (PMC8144621; doi:10.1038/s41598-021-90003-1)
Supplement: Supplementary file 3 — 3. Supplementary Information [file 41598_2021_90003_MOESM3_ESM.pdf]

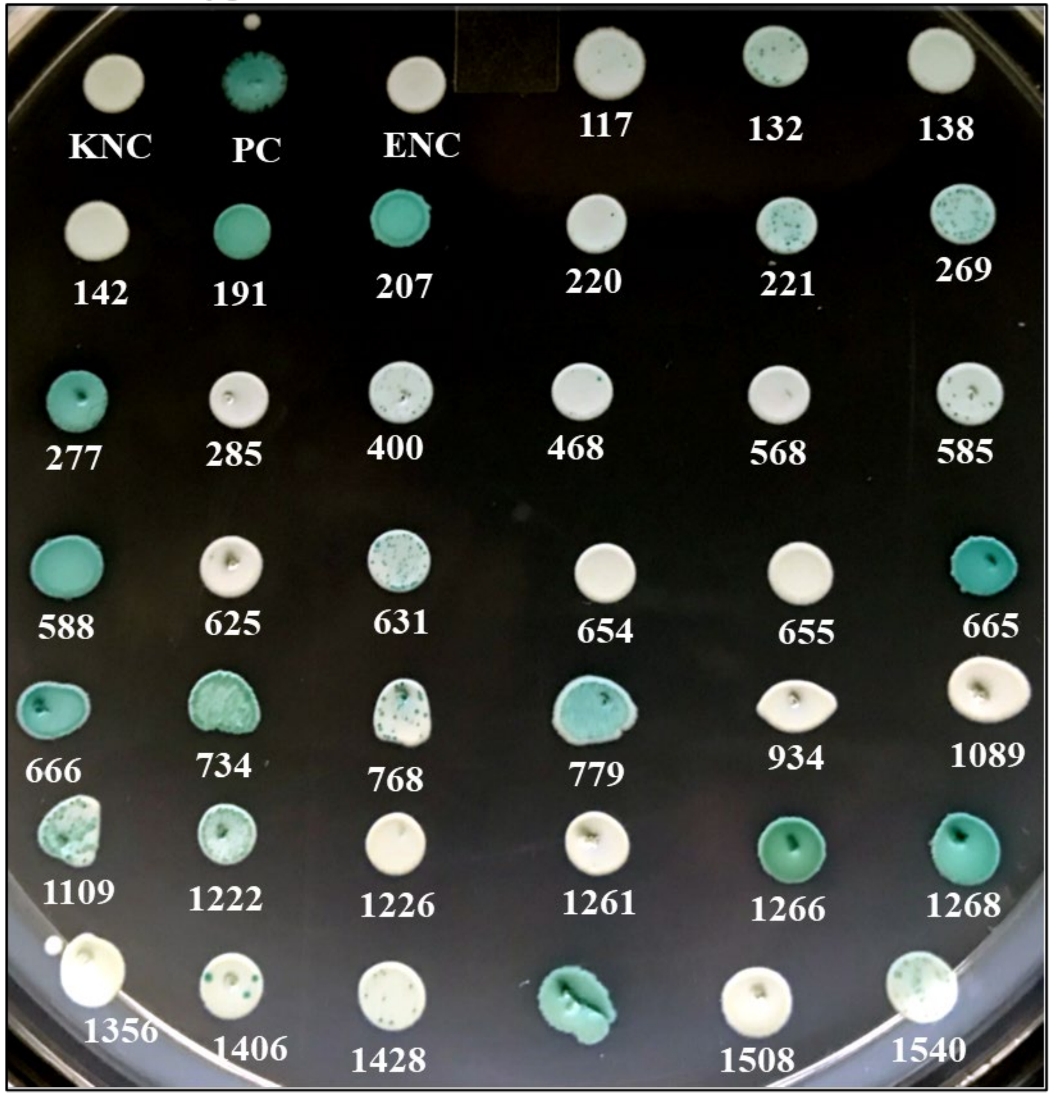

Protein 1: T25-**HP0207(ApbC)**$^{\mathbf{WT}}$
Protein 2: T18-**HP# (gene number, strain 26695)**

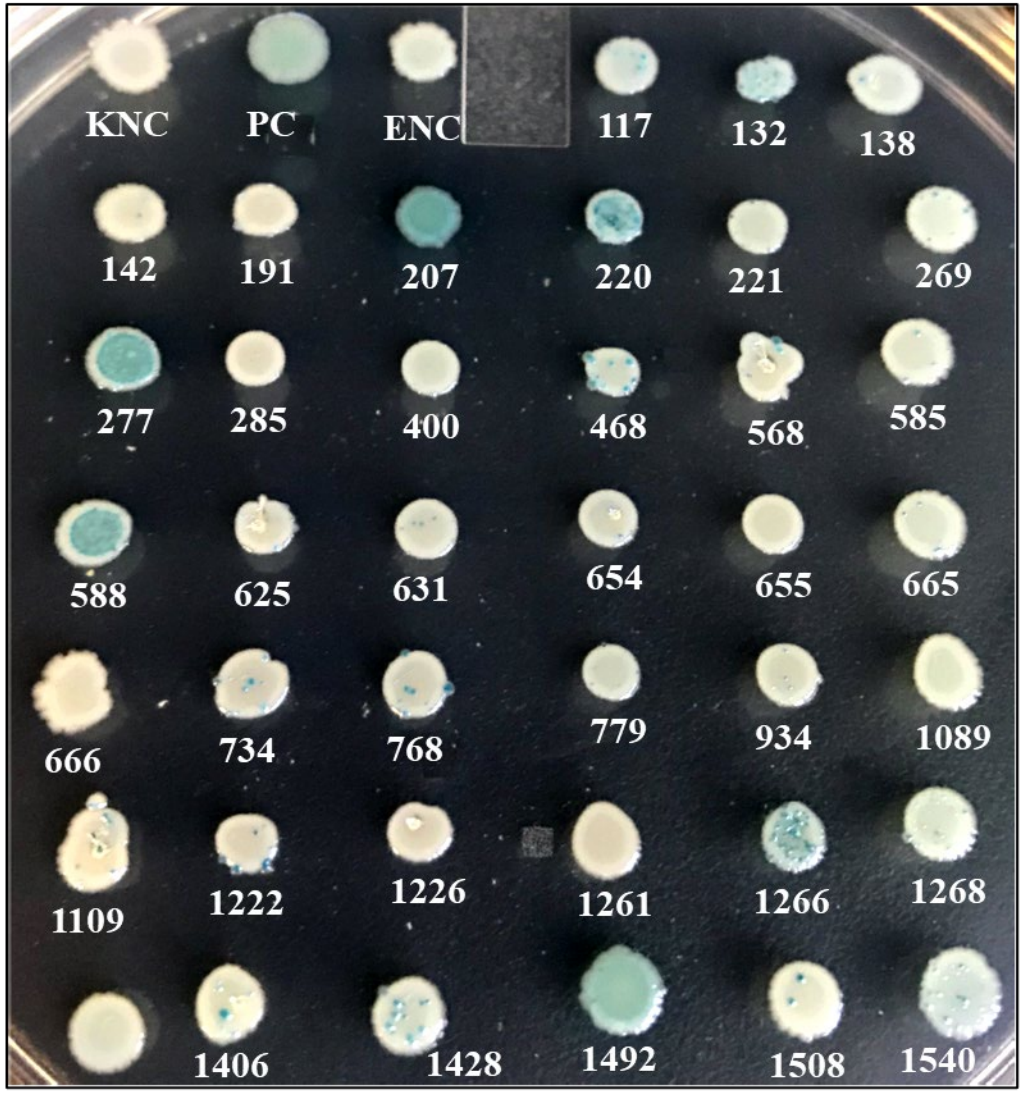

Protein 1: T25-**HP0207(ApbC)**$^{\mathbf{K106A,K111A}}$
Protein 2: T18-**HP# (gene number, strain 26695)**

Supplemental Fig. S2 for Benoit, Agudelo and Maier, Scientific Reports, 2021.
Pictures of Mac Conkey maltose medium showing interactions between *H. pylori* ApbC^{WT} and Fe-S proteins (left panel) or ApbC $^{\mathbf{K106A,K111A}}$ and Fe-S proteins (right panel).
A volume of 1 µL of *E. coli* cells expressing plasmid-borne *H. pylori* fusion proteins (as indicated underneath each plate) was spotted on LB-X-Gal. Cells were incubated at 30°C for 36-48 h under aerobic conditions. Clones positive for protein-protein interaction turned blue. **KNC**: Kit Negative Control: T18 + T25. **PC**: Positive Control: T18-zip + T25-zip.
ENC: Experiment Negative Control: T18 + (T25-HP0207^{WT} or T25-HP0207$^{K106A, K111A}$).
