# Supplementary material for: A two-hybrid system reveals previously uncharacterized protein–protein interactions within the Helicobacter pylori NIF iron–sulfur maturation system
Source: Sci Rep. 2021 May 24;11:10794. doi: 10.1038/s41598-021-90003-1 (PMC8144621; doi:10.1038/s41598-021-90003-1)
Supplement: Supplementary file 4 — 4. Supplementary Information [file 41598_2021_90003_MOESM4_ESM.pdf]

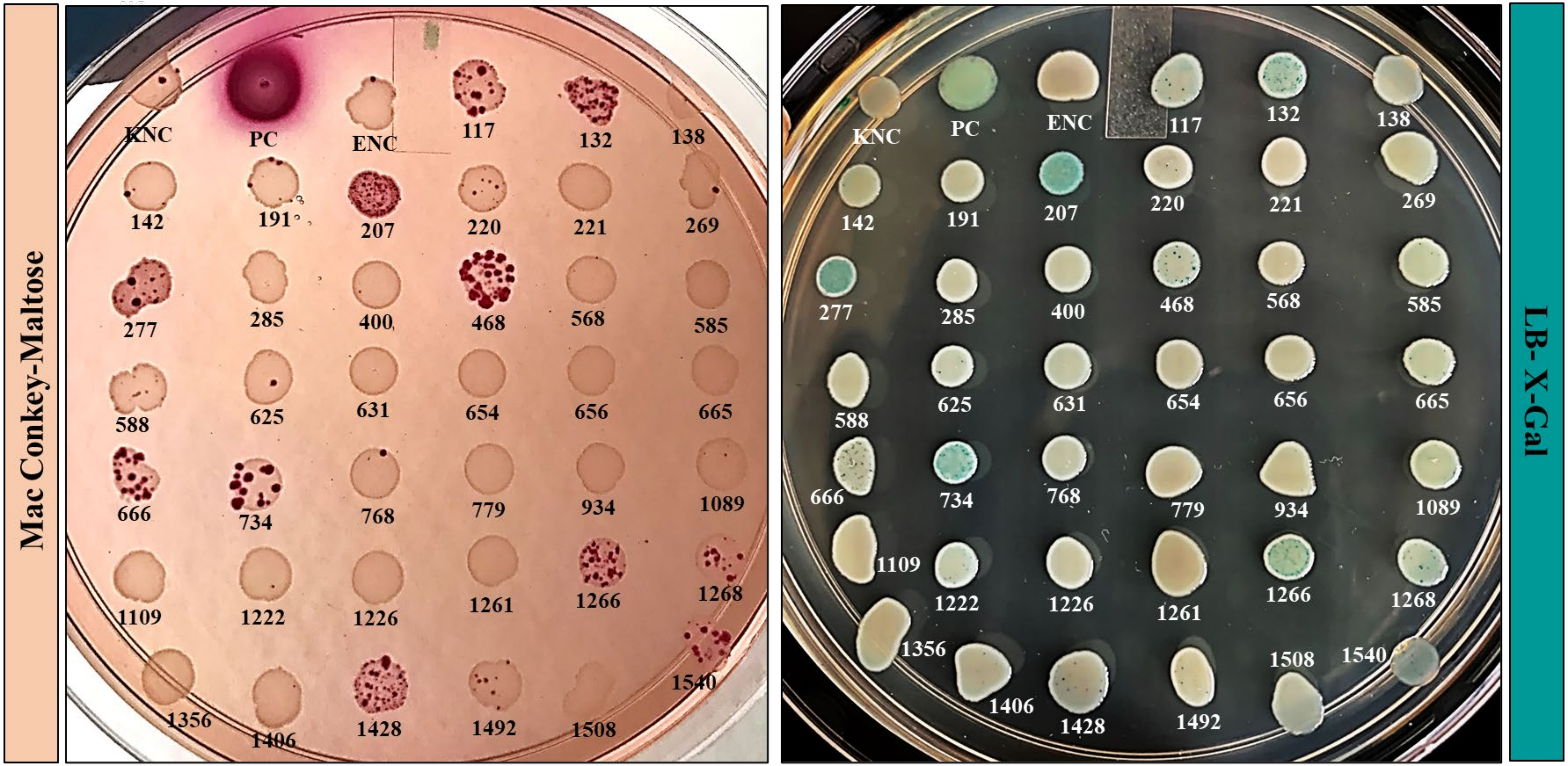

Protein 1: T25-**HP0277(FdxA)**
Protein 2: T18-**HP# (gene number, strain 26695)**

Protein 1: T25-**HP0277 (FdxA)**
Protein 2: T18-**HP# (gene number, strain 26695)**

Supplemental Fig. S3 for Benoit, Agudelo and Maier. Scientific Reports, 2021.
Pictures of Mac Conkey maltose (left) or LB-X-Gal medium (right) showing interactions between *H. pylori* HP0277 (FdxA) and various Fe-S proteins.
A volume of 1 μL of *E. coli* cells expressing plasmid-borne *H. pylori* fusion proteins (as indicated underneath each spot) was spotted on MC Mal (left panel) or LB-X-Gal (right panel) .
Cells were incubated at 30°C for 36-48 h under aerobic conditions. Clones positive for protein-protein interaction turned red on MC Mal (left panel) and blue on LB-X-Gal (right panel).
KNC: Kit Negative Control: T18 + T25. **PC**: Positive Control: T18-zip + T25-zip. **ENC**: Experiment Negative Control: T18 + (T25-HP0207^{WT} or T25-HP0207$^{K106A, K111A}$)
